# Effects of beetroot juice intake on repeated performance of competitive swimmers

**DOI:** 10.3389/fphys.2022.1076295

**Published:** 2023-01-10

**Authors:** Berta Moreno, Esther Morencos, Davinia Vicente-Campos, Alejandro Muñoz, Jaime González-García, Santiago Veiga

**Affiliations:** ^1^ Facultad de Ciencias de la Salud, Universidad Francisco de Vitoria, Pozuelo de Alarcón, Spain; ^2^ Departamento de Deportes, Universidad Politécnica de Madrid, Madrid, Spain

**Keywords:** swimming, nitrate, beet, ergogenic aids, intermittent sports

## Abstract

**Background:** Beetroot juice is a sport supplement with a high level of evidence on the physical performance enhancement. However, in swimming, there is no clear data about the effects of beetroot juice on performance.

**Objective:** To investigate whether an acute intake of beetroot juice (BJ) improves the performance of competitive swimmers in a repeated maximum swimming effort.

**Method:** Thirteen national-level swimmers (six females and seven males), participated in this randomized, double-blind crossover study. In two different trials, swimmers ingested a 70-mL placebo shot (.04 mmol NO_3_^−^; PLA) or a 70-mL Beet-It shot (6.4 mmol of NO_3_^−^beet juice [BJ]) 3 h before undergoing a 6 × 100-m front-crawl maximal effort test with 7 min rest between each 100 m.

**Results:** Overall, 100-m times showed no difference between the BJ and PLA groups (*p* = .364), although a possibly shorter time was observed for BJ in the last repetition (*p* = .104; mean difference [MD] = −.99 s, mean-based inference [MBI] = 49/51/0). Participants in the BJ condition showed a possibly lower rate of perceived exertion in the first (*p* = .242, MD = −.85, MBI = 70/28/2) and second repetitions (*p* = .165, MD = 1.15, MBI = 83/16/1), whereas Total Quality Recovery scale scores were likely higher in the first (*p* = .110, MD = 1.15, MBI = 83/16/1) and third (*p* = .082, MD = −.77, MBI = 70/29/1) repetitions compared with those in the PLA group. Blood lactate concentration [La^+^] levels showed no differences between groups in any of the repetitions (*p* > .05, unclear), and we observed an increase in 100-m times for both BJ and PLA (BJ: *p* = .014, MD = −1.51 s; PLA: *p* = .029, MD = −1.57 s) after the fifth repetition.

**Conclusion:** No clear differences in performance were observed in a 6 × 100-m repeated sprint test by competitive swimmers when supplementing (or not) with BJ. However, there was a trend toward a better recovery between efforts and a better tolerance of fatigue when swimmers ingested BJ.

## 1 Introduction

Swimming is a sport in which most competitive events range from 20 s to 4 min and in which swimmers must overcome the drag forces of the water to accelerate their center of mass forward. This makes strength and power production, as well as technical competence (Derave and Tipton, 2014; Muniz-Pardos et al., 2019; Muniz-Pardos et al., 2020), some of the main determinants of performance. The schedules of major swimming competitions (i.e., Olympic Games or World Swimming Championships) are arranged for multiple participation of athletes; most events consists of two or three rounds (heats, semifinals, and finals) and the races are distributed over the course of 6 to 8 days.

The characteristics of swimming competitions somehow reflect the characteristics of elite training programs in that, even for the shorter events, they are characterized by high volumes of annual in-water training (≈2,000–2,500 km) and training devoted to the 2 to ≤4 mmol·L-1 training zone (≈44–45% of total volume) (Hellard et al., 2019). The aim of this preparation would be to highlight the athletes’ swimming technical efficiency (Hellard et al., 2019) and to prepare swimmers for repeated performances at swimming events; it would include double training sessions (i.e., morning and afternoon) or a variety of intervallic training methods with efforts repeated under different recovery durations. As a result of these training characteristics, athletes including competitive swimmers usually use sports supplements and nutritional support to obtain potentially valuable contributors to success (Maughan et al., 2018). At the 2,000 Olympics, 99% of swimmers used supplements (including sports foods), and 94% used at least one non-food dietary supplement (Shaw et al., 2014). In addition, Shaw et al. (2016) identified that 97% of elite Australian swimmers reported taking supplements or sports foods during a 12-month training period. At present, 86.9% of elite swimmers have been reported to consume sports supplements, with no differences observed between men and women (Moreno et al., 2022).

In order to classify nutritional supplements according to their likelihood to provide a detectable benefit, the Australian Institute of Sport (2022) created a system for classifying supplements in different groups, from Class A (permitted and most likely to be beneficial according to evidence) to Class D (prohibited). Beetroot juice (BJ), which is included in the Class A group, has been found to have a high concentration of inorganic nitrate (NO_3_^−^), and there is evidence that NO_3_^−^ supplementation improves vasodilation and increases blood flow in muscle (Jones et al., 2018; McDonagh et al., 2019), specifically on the type II muscle fibers (Ferguson et al., 2013; Van De Walle and Vukovich, 2018; Jodra et al., 2019; Senefeld et al., 2020; Zamani et al., 2020). In addition, BJ intake has been found to increase the release and subsequent reuptake of calcium from the sarcoplasmic reticulum in mice (Hernández et al., 2012). This could translate into a greater muscle force production capacity of these type II muscle fibers and, therefore, into a physiological advantage for high-intensity intermittent efforts (Domínguez et al., 2018), recovery in a repeated effort, and preservation of power over several repetitions (Rojas-Valverde et al., 2021). For example, a study conducted by (Clifford et al., 2016) demonstrated an improvement in physical performance measured by countermovement jumping when supplementing with BJ. The improved blood flow during physical exercise could also contribute to the decrease in the subjective perception of effort (Jodra et al., 2019) and could be effective in situations where oxygen availability is less (Kelly et al., 2014; Flueck et al., 2016), as occurs in swimming, especially during the underwater sections (Veiga et al., 2022). However, very little research has explored the benefits of BJ supplementation in competitive swimmers, especially in relation to the repeated efforts that characterize swimming competitions.

To our knowledge, only three studies have examined the possible benefits of BJ consumption in swimmers (Pinna et al., 2014; Lowings et al., 2017; Esen et al., 2018), but none of them have yielded clear results in regard to performance. Certain physiological parameters, such as oxygen consumption (VO_2_), may be hampered within the aquatic environment (Pinna et al., 2014) and the stringency of the intake protocol for BJ (Senefeld et al., 2020) may somehow limit research on this supplemental aid for swimming. Some other limitations of the existing studies, such as sample consisting of only moderately trained swimmers (Derave and Tipton, 2014; Senefeld et al., 2020), testing performed over non-competitive distances (Esen et al., 2018; Ruiz-Navarro et al., 2022), the lack of control group (Esen et al., 2018), and differences in the supplementation protocol (acute or chronic), likely have prevented researchers from drawing solid conclusions. In this context, the aim of our study was to investigate whether an acute intake of BJ improved the performance of competitive swimmers in a repeated maximal swimming effort. We hypothesized that competitive swimmers ingesting BJ would not display an overall better performance compared with a control group but that they would reveal an improvement in the psychophysiological parameters, improving the level of repeated effort.

## 2 Materials and methods

### 2.1 Participants

Thirteen competitive swimmers six females (15.3 ± 1.75 years old, 1.63 ± .08 m, 55.41 ± 8.76 kg, and 20.90 ± 2.74 body mass index) and seven males (16.4 ± 1.4 years old, 1.76 ± .03 m, 63.02 ± 6.46 kg, and 20.22 ± 1.66 body mass index) participated in this study. They were part in a training plan of at least 18 h/wk in the water (approximately 40–50 km/wk), 5 h/wk of out-of-water training and had competitive experience of at least 4 years. Their average personal best times corresponded to Level 3 (national level), according to Ruiz-Navarro et al. (2022). Written informed consent was obtained from all participants and their tutors after we informed them of the method and purpose of the study. The study design and protocol were approved by the local university ethics committee (No. 42-2021) and complied with the recommendations of the Declaration of Helsinki.

### 2.2 Experimental design

The experiment was randomized, placebo-controlled, double-blinded, and balanced to eliminate any order effects. The ClinicalTrials.gov identifier was NCT05470231. Each participant performed two sessions separated by 18 days to allow for recovery and substance washout, in their usual training pool and under the same experimental conditions (i.e., same time of day). The trial was double-blinded, so that an external investigator assigned all participants’ drinks in a counterbalanced fashion with random assignment to each supplement (Research Randomizer, https://www.randomizer.org). In the first session, seven swimmers ingested the BJ supplement and six ingested a placebo (PLA) whereas, in the second session, six swimmers ingested the BJ supplement and seven ingested a placebo (PLA).

### 2.3 Procedure

**FIGURE 1:**
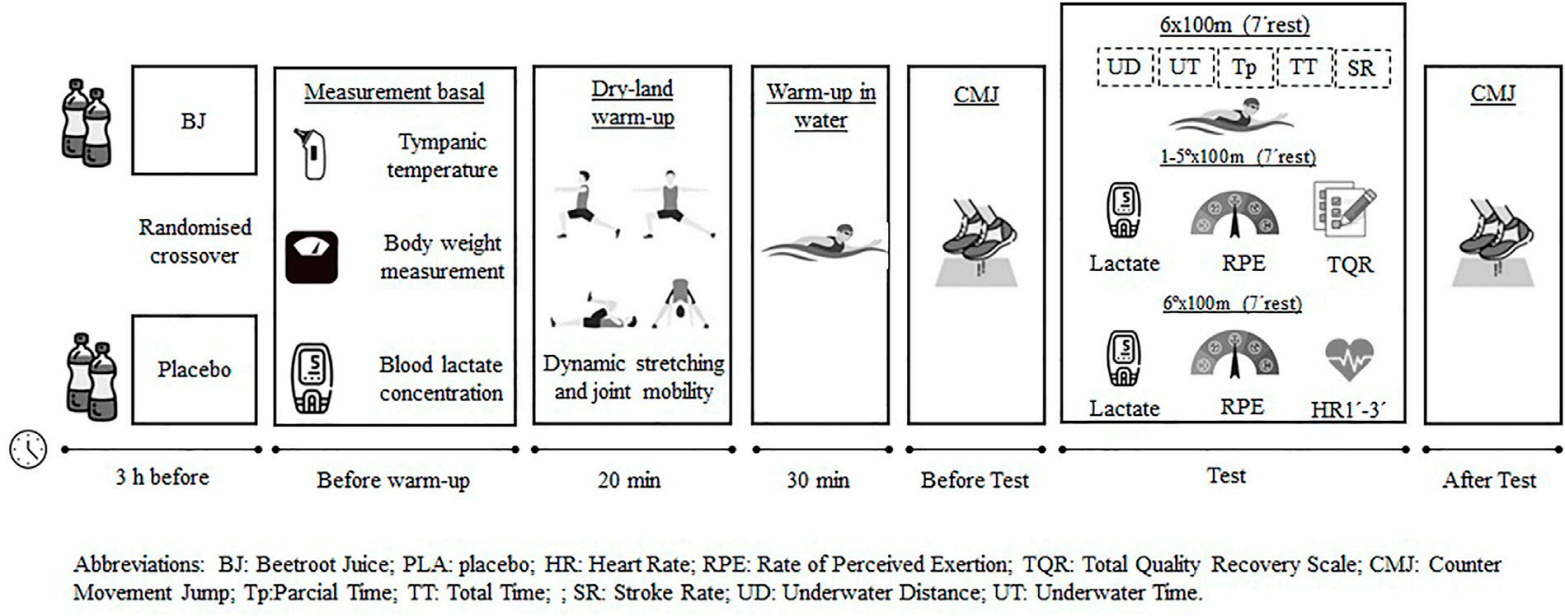
Diagram of the intervention process.

After a morning of fasting, the swimmers arrived at their usual training pool 3 h before the performance test (see Figure 1). On arrival at 7:30 a.m., they were provided with a nitrate shot containing 70 mL dose of beetroot juice whith 6.4 mmol of NO_3_^−^(Beet-It-Pro Elite Shot, James White Drinks Ltd., Ipswich, United Kingdom) or 70 mL nitrate-depleted beetroot juice placebo (.04 mmol of NO_3_^−^), matched in flavour, appearance, and packaging (Beet-It-Pro Elite Shot, James White Drinks Ltd., Ipswich, United Kingdom), as described elsewhere (Domínguez et al., 2017; Senefeld et al., 2020). At the end of both trials, to determine whether they had identified on which day they had ingested the BJ and or PLA (López-Samanes et al., 2020) they were asked “What do you think you ingested?” On the first day, four participants guessed correctly what they had drunk and on the second day only one participant guessed correctly. All participants were instructed to follow a diet protocol the day before that consisted of 60% carbohydrate, 30% fat, and 10% protein (Jodra et al., 2019), and a list of foods rich in NO (e.g., beets, celery, spinach) was provided to them that they were to eliminate from their diet in the 48 h before each trial (Miraftabi et al., 2021). In addition, in the 24 h before each trial, swimmers were encouraged to avoid brushing their teeth or using any oral rinses; using chewing gum; and ingesting sweets, stimulants (e.g., caffeine), or alcohol, all of which could alter the oral microbiota and interfere with NO_3_^−^ reduction.

After the sports supplements intake, we measured the swimmers’ body composition (Tanita B-601; Tanita Corp, Tokyo, Japan) and tympanic temperature (Thermo-Scan 7-IRT 6520; Braun, Frankfurt, Germany) to ensure similar conditions before each trial, as in other studies (Muñoz et al., 2020), and we measured their resting lactate using a Lactate Pro2 portable lactate analyzer (Arkray, Shiga, Japan). A record sheet was used to record what they had eaten for dinner the night before, so that in the next trial they could be reminded of what they had to eat for dinner again.

After resting values had been measured, the swimmers did a standardized dry-land warm-up consisting of dynamic stretching and joint mobility for 20 min. Afterward, the swimmers performed another standardized warm-up in water for about 30 min, including some technique drills and descending repetitions. After the pool warm-up, swimmers completed three repetitions of Counter Movement Jump (CMJ) with hands on waist and with 45 s of passive recovery, using an infrared jumping system (Optojump Next; Microgate, Bolzano, Italy) The highest height of the three jumps was recorded.

Three hours after having ingested the BJ or PLA, the main test, consisting of six repetitions of 100-m front crawl at maximum effort from a dive start and with 7 min of passive recovery between each repeat was performed. The swimmers performed the test one by one, and two experienced coaches, whose times were averaged, took 50-m split times and 100-m total times using a Finnis Chronometer 3 × 100 digital stopwatch. One minute after completing each test repetition, blood lactate concentrations were measured with the portable Lactate Pro2 analyzer. Around 30 s after the lactate measurements, for each swimmer a rating of perceived exertion (RPE) was used with the Borg CR-10 category-ratio scale (Borg, 1982). In addition, and just before the next 100-m repetition, swimmers were asked about their recovery state using the Total Quality Recovery Scale (TQR) (Kenttä and Hassmén, 1998). Immediately after completing the last repetition of 100 m, each swimmer’s heart rate was measured for a period of 30 s using a Geonaute Oxylane-4 heart rate monitor. Finally, after the last 7-min break, the swimmers performed three CMJ jumps using the same procedure as before the 6 × 100-m test.

### 2.4 Data processing

The entire 6 × 100-m test was recorded at 25 Hz with a fixed JVC Model GY-HM150E video camera located on the public stands at 12.5 m from the start or turn wall and 5 m above and 10 m away from the swimmers’ lane. Using the colored buoys from floating lanes, video images were calibrated using Kinovea (Version 9.5; Joan Charmant & Contrib.) to calculate the distance and time spent by swimmers during the underwater sections of start and turns. In addition, from the video footage the swimmers’ stroking rates (cycles/min) on the second or third lap of each repetition were collected.

### 2.5 Statistical analysis

Statistical tests were carried out using IBM SPSS Statistics for Macintosh (Version 26.0; IBM Corp., Armonk, NY). We performed a Shapiro–Wilk test to determine whether the distribution of the data met the normality assumption and used a two-way repeated-measures analysis of variance to identify the effects of the BJ or PLA on the dependent variables. Variance and sphericity assumptions were checked with Levene and Mauchly tests, respectively. Partial eta-squared (η_p_^2^) values (classified as follows: small = .01, medium = .06, large = .14) (Cohen, 1992) were calculated, and a Bonferroni’s *post hoc* test was used to check pairwise comparisons. In addition, estimated magnitudes (Cohen’s *d*, 90% confidence intervals [CIs]) were calculated between the PLA and BJ conditions to allow a magnitude-based decision approach. We used log-transformed data to reduce non-uniformity error bias. The smallest significant standardized effect threshold was set as .2. Ranges of likelihood <1% indicated almost certainly no chance of change; 1% to 5%, very unlikely; 5% to 25%, unlikely; 25% to 75%, possible; 75% to 95%, likely; 95% to 99%, very likely; >99%, most likely. Differences were rated as unclear when likelihood exceeded 5% in both positive and negative directions. Estimated magnitudes were classified in standardized units as follows: ≤.2 is trivial, ≥.2–.6 is small, ≥.6–1.2 is moderate, ≥1.2–2.0 is large, and ≥2 is very large (Hopkins et al., 2009). Results are expressed as *M* ± *SD*. The significance level was set at *p* < .05.

## 3 Results

**FIGURE 2:**
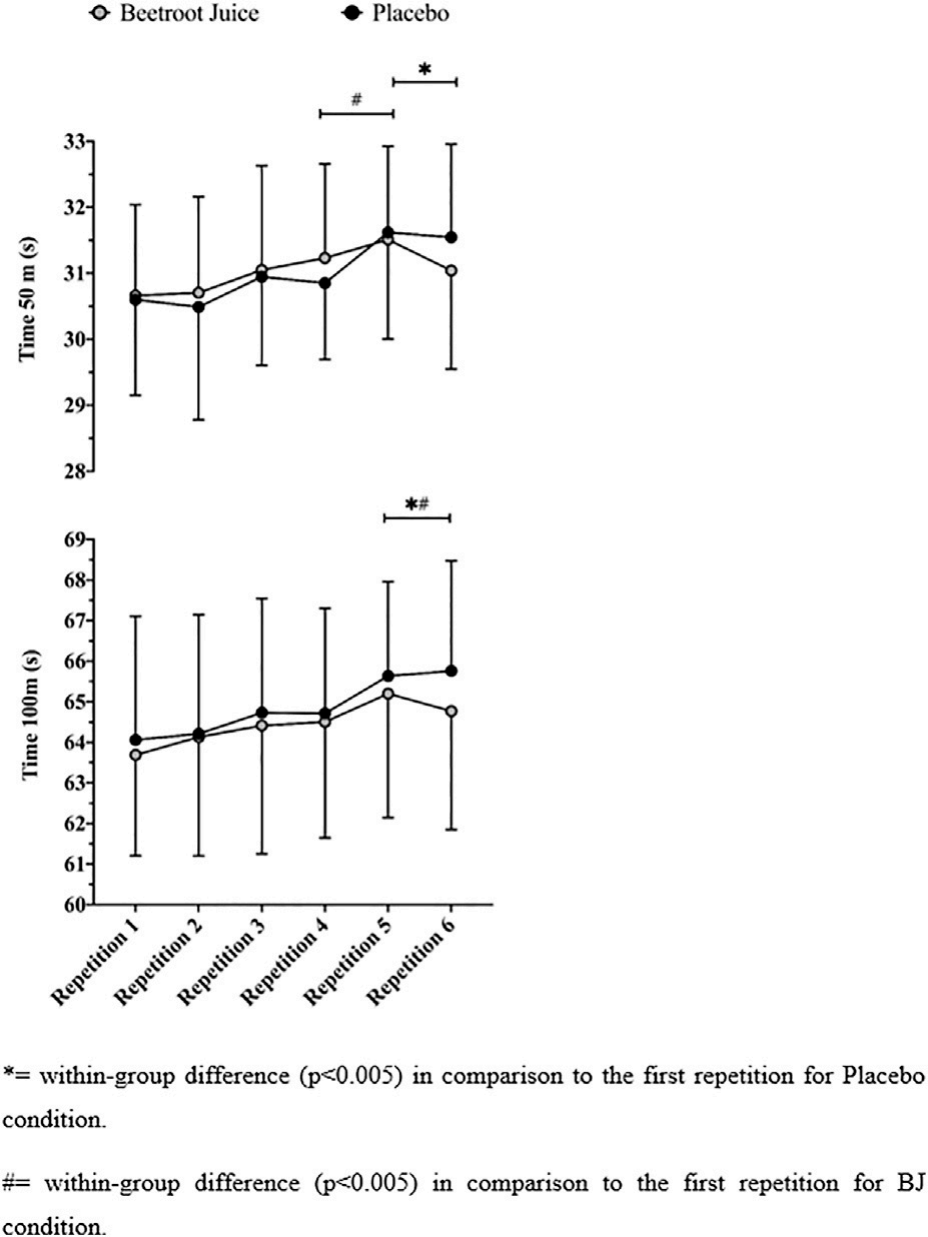
Fifty- and 100-m repeated sprint performance for the beetroot juice (BJ) and placebo conditions.

Overall, no differences were observed in any of the interactions (repetition * condition) of the variables analyzed (*p* > .05). Attending to pairwise comparisons, no differences in partial (50-m) or total (100-m) times (see Figure 2) were observed between the PLA and BJ conditions in any of the six repetitions (*p* > .05; effect size [ES] = −.14–.20), although a small possibly positive effect of BJ was detected on the partial (*p* = .112; mean difference [MD] = −.51 s, [95% CI] [−1.15, .14]; *d* = .20, [90% CI] [−.01, .41]; mean based inference [MBI] = 49/51/0) and total (*p* = .104; MD = –.99 s, [95% CI] [−1.15, .14]; *d* = .20, [90% CI] [−.01 to .40]; MBI = 49/51/0) times of the last repetition. Compared with the first repetition, longer lengths in the partial times were observed for the PLA group in the fifth and sixth repetitions (*p* = .025; MD = −.57 s, [95% CI][−1.05, −.09]) and for the BJ in the fourth (*p* = .024; MD = −.57 s, [95% CI] [−.89, .12) and fifth (*p* = .009; MD = −.85 s, [95% CI] [−1.44, −.09]) repetitions. Similarly, slower 100-m times were observed after the fifth repetition for both groups (BJ: *p* = .014; MD = −1.51 s, [95% CI] [−2.65, −.37]; PLA: *p* = .029; MD = −1.57 s, [95%CI] [−2.96, −.19]).

**FIGURE 3:**
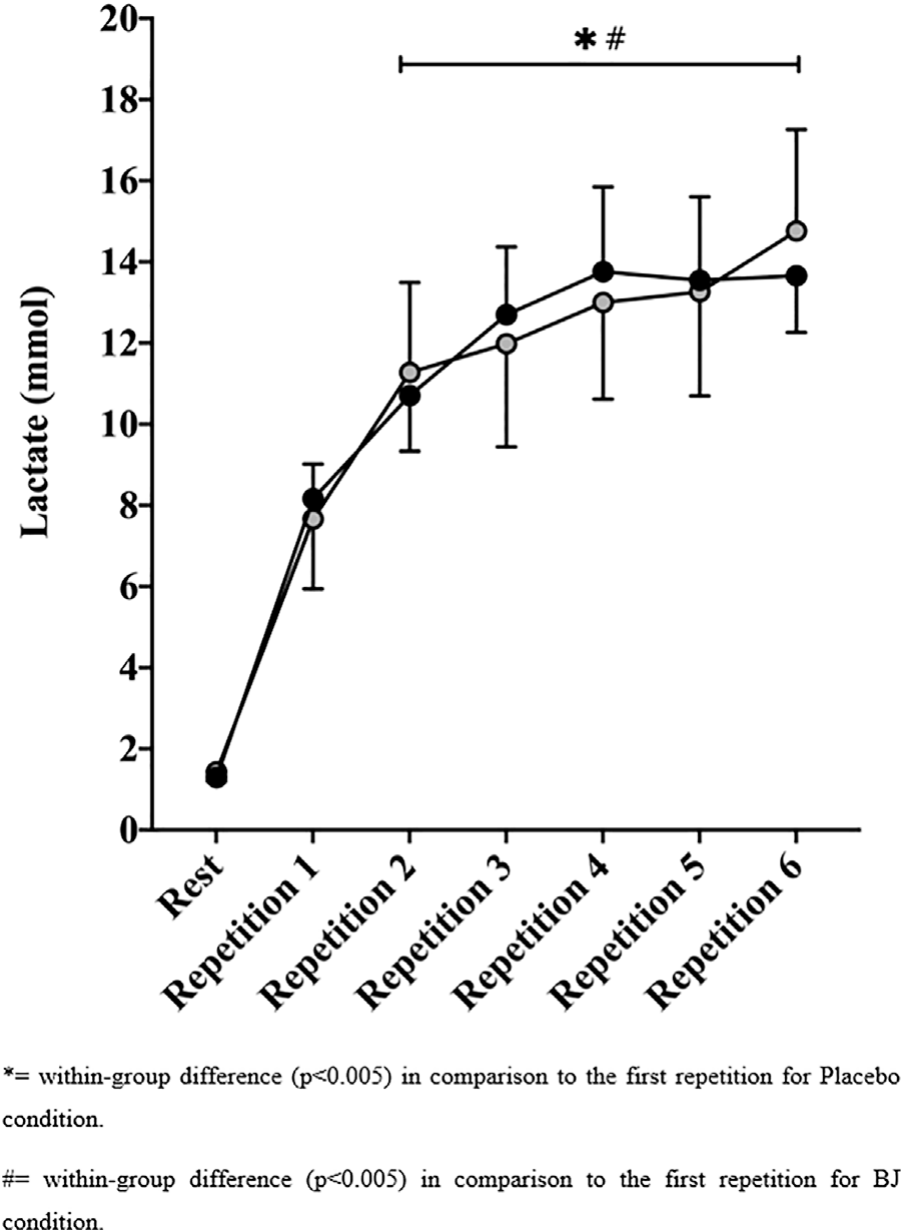
Lactate response at rest and after each 100-m sprint repetition for the beetroot juice (BJ) and placebo conditions.

**FIGURE 4:**
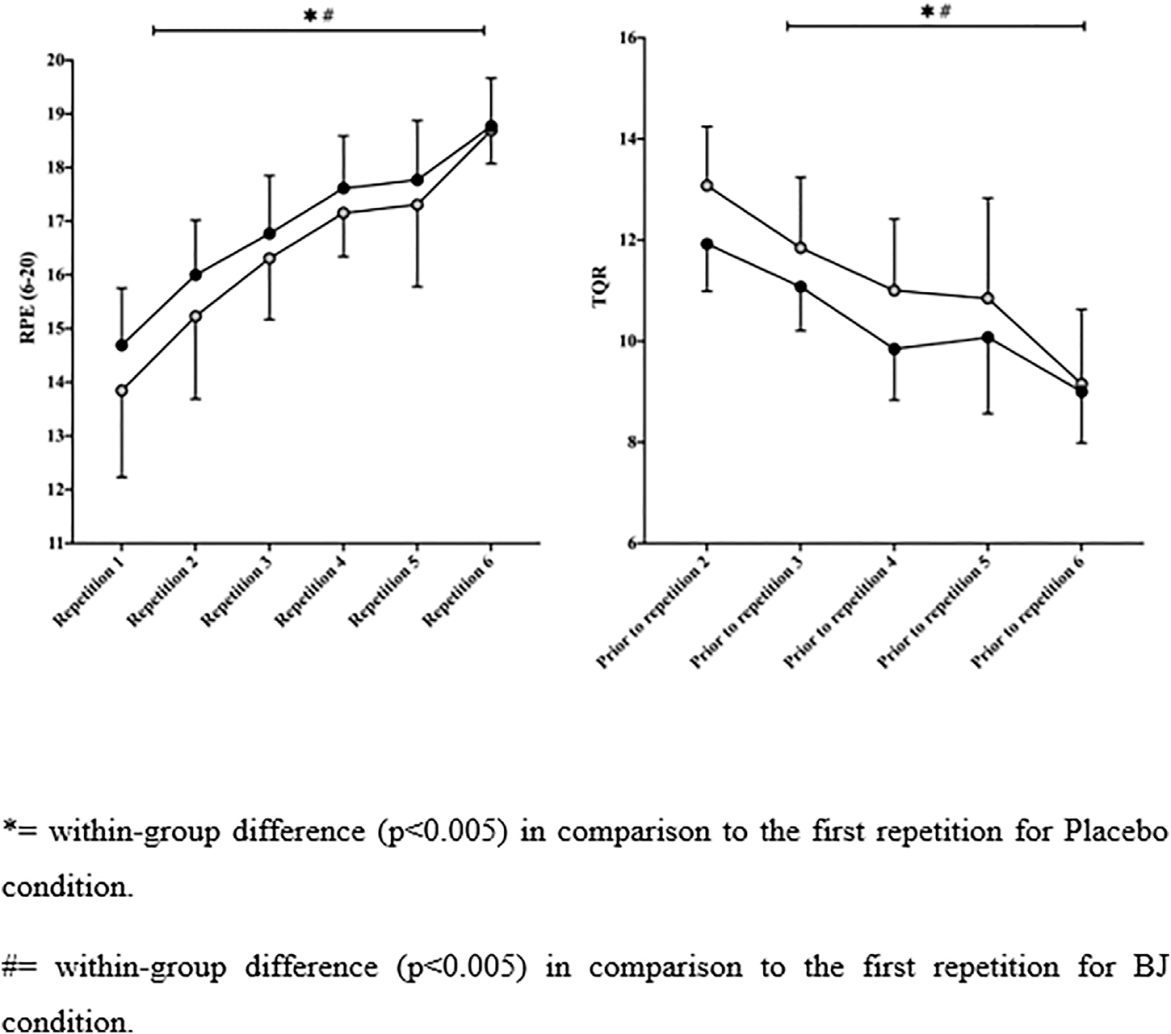
Rating of perceived exertion (RPE) and total quality recovery scale (TQR) responses after each 100-m sprint repetition for the beetroot juice (BJ) and placebo conditions.

Blood lactate concentrations (see Figure 3) were similar between the PLA and BJ conditions after each of the six repetitions (*p* > .05; ES = −.34–.24). The evolution of blood lactate levels showed an increased concentration after each repetition in both conditions PLA (*p* < .001, MD = 6.23–13.21 mmol) and BJ (*p* < .001, MD = 6.87–2.37 mmol). There were no differences between conditions in RPE (*p* > .05; ES = .04–.33) and TQR (*p* > .05; ES = −.53–.00) after each of the six repetitions. However, the BJ condition showed a small possibly positive effect in RPE in the first (*p* = .242; MD = −.85 s, [95% CI] [−2.34, .65]; *d* = .33, [90% CI] [−.10, .70]; MBI = 70/28/2) and second repetitions (*p* = .165; MD = 1.15 s, [95% CI] [−.17, 2.48]; *d* = .44, [90% CI] [.01 to .87]; MBI = 83/16/1), whereas TQR values were likely higher in the first (*p* = .110; MD = 1.15 s, [95% CI] [−.30, 2.61]; *d* = .53, [90% CI] [−.05, 1.12]; MBI = 83/16/1) and third (*p* = .082; MD = −.77 s, [95% CI] [−1.90, .36]; *d* = .31, [90% CI] [−.04, .65]; MBI = 70/29/1) compared with the PLA condition. The evolution of RPE and TQR values indicated increased values from the first repetition in both groups (see Figure 4). No differences between groups were observed for maximum heart rate or CMJ after the 6 × 100 m (*p* > .05).

The underwater distance on the start was greater in the BJ condition in the first (*p* = .034; MD = .34 m, [95% CI] [.03, .64]; *d* = .44, [90% CI] [.13, .75]; MBI = 90/10/0) and fourth (*p* = .0014; MD = .38 m, [95% CI] [.09, .66]; *d* = .34, [90% CI] [.13, .55]; MBI = 87/13/0) repetitions. In contrast, no difference was observed between conditions for the turn underwater distance in any of the repetitions (*p* > .179; ES = .01–.30). For the BJ condition, the evolution of underwater distance indicated a greater start distance in the fourth repetition than in the first (*p* = .009; MD = .49 m, [95% CI] [.15, .83]) and second (*p* = .004; MD = .41 m, [95% CI] [.15, .66]). For the PLA group, the start underwater distance of the third repetition was longer than the first one (*p* < .028; MD = .43–.51 m). Values of underwater velocity on the start (*p* > .149; ES = −.03–.47) and turn (*p* > .264; ES = −.16–.32) segments were similar in the BJ and PLA groups for all repetitions. BJ presented a small likely positive effect over PLA in the start underwater velocity of the first repetition (ES [95% CI] = .47 [−.12, 1.06] MBI = 79/18/3). No more within-group differences were observed for both underwater velocities (*p* > .05). Similarly, there were no significant differences in stroke frequency across repetitions (*p* = .704) or between the different conditions (*p* = .407).

## 4 Discussion

In this study, we aimed to evaluate whether an acute intake of BJ affected the repeated performance of competitive swimmers as well as their physiological, kinematical, and psychophysiological parameters. In agreement with our hypothesis, BJ did not generally improve the swimming performance over a PLA in the six maximal swimming efforts. The BJ supplementation appeared to have a greater effect on the power generated in the 50 and 100-m times in the sixth (last) repetition (Figure 2), but the improvements observed were not significant.

Although there is lack of evidence about tests of time and intensity similar to those carried out in the present research, Franchini et al. (2016) evaluated the participation of the energy systems in four successive Wingate tests performed with upper limbs and with 3 min of passive recovery. The main findings of that study showed how the contribution of oxidative metabolism remained constant through the four tests. This could explain why the possible beneficial effect of BJ supplementation was not observed in the present study, considering the greater ergogenic potential of BJ on the oxidative pathways (Franchini et al., 2016). An understanding of the energy system contributions to upper body sprint interval exercises is only beginning and need more investigation.

The lack of overall differences in swimming performance between the BJ and PLA groups is consistent with previous research on maximal efforts at similar competitive distances of 100 m and 200 m but with a 3-day BJ supplementation period (Esen et al., 2018). However, the potential improvement of the BJ group in the fifth and sixth 100-m efforts would be in line with a previous study that detected possible beneficial effects of BJ on the second half of a 8 × 21-m effort (Lowings et al., 2017). Perhaps the improvement of muscle efficiency and contractile capacity by promoting the release of calcium from the sarcoplasmic reticulum and its reuptake into muscle cells (Bloomer et al., 2010; McMahon et al., 2017) could help increase the strength of muscle contraction in the last repetitions.

The observed blood lactate concentrations in all repetitions were higher than the resting values in both the PLA and BJ conditions, with no differences between them. These results support those of previous studies with swimmers in which no positive effect of BJ intake on lactate concentration was demonstrated (Lowings et al., 2017; Esen et al., 2018), and they are in line with previous results in other sport disciplines based on high-intensity intermittent exercise (Balsalobre-Fernández et al., 2018; Miraftabi et al., 2021). However, previous studies have obtained lower levels in blood lactate in recreational runners when using a chronic NO_3_^−^ supplementation during a 4-week period (Santana et al., 2019), which probably is related to a change in the energy supply from an anaerobic source to an oxidative one (Bloomer et al., 2010). It could be that the effects of NO_3_^−^ supplementation on blood lactate rely more on a chronic supplementation period/protocol rather than on an acute intake.

Swimmers’ RPE and TQR values in this study were similar between conditions during the six efforts and their associated recovery periods. Only a probable small perceived better recovery was observed for BJ in the first and third repetitions that could be related to the lower perceived effort in the first repetition (Figure 4). These results partly contradict those found in a group of elite athletes who experienced lower RPE values in a single incremental test after 15 days of BJ intake (Balsalobre-Fernández et al., 2018) and those of resistance trained students with a decreased postexercise RPE following acute BJ supplementation before a Wingate test (Jodra et al., 2019). The increase in blood flow to the frontal lobe of the brain (Presley et al., 2011), where the emotions and decision-making processes are regulated, could contribute to the subjective integration of perceived exertion (Williamson et al., 2001). In addition, the increased cerebral blood flow may have contributed to a probable small perception of a better RPE and TQR, given that reduced blood flow to the brain during exercise is related to the onset of fatigue (Oggioni et al., 2018).

The maximum heart rate values did not show significant differences between groups and, according to the results of the RPE scale on the last repetition, 18.77 ± 1.48 for PLA and 18.69 ± 1.03 for BJ, we can confirm that the efforts of our swimmers were maximal in both conditions. Even so, our results are in line with those of other studies that used a single dose of BJ (Cermak et al., 2012) or several doses (Levitt et al., 2015; Oggioni et al., 2018) and also found no effect of BJ on heart rate. The CMJ performance was also included as a measure of strength performance, but the BJ did not improve countermovement jumping performance, as indicated in previous research in both recreational and elite athletes (López-Samanes et al., 2020; Jonvik et al., 2021) or even when combining BJ supplementation with caffeine (Berjisian et al., 2022). Jumping performance is determined, among others, by the contractile properties of the muscle and by the neuromuscular control of the entire musculoskeletal system (Bobbert and Knoek van Soest, 2001). In the same line, no effects of BJ supplementation were observed in the swimming stroke frequency of the participants, which is also mainly determined by the swimmers’ strength-related ability (Morais et al., 2018).

In relation to the underwater sections of swimming efforts, the swimmers in the present study did not show large differences between the two conditions, with some improvement in the distances and average speed of the first and fourth repetition starts in the BJ condition. It has previously been reported that supplementation with BJ could have positive effects on underwater swimming performance by divers (Engan et al., 2012) because supplementation can assist in situations in which oxygen availability is less (Kelly et al., 2014; Flueck et al., 2016). However, both our results and those of Lowings et al., (2017) suggest that BJ supplementation does not particularly affect the contribution of underwater swimming to the swimming efforts of competitive swimmers. The fact that underwater distances by national-level swimmers in the present study were shorter than previously reported in 100 and 20-0 m front-crawl competitions (Veiga et al., 2014) probably indicates that the participants did not obtained the full potential of these underwater segments, and this could hinder BJ effects at this point.

The present findings provide interesting insights for coaches and professionals involved in competitive swimming because it is well known that time differences at the elite level are becoming increasingly narrow (Born et al., 2022) and that marginal gains may represent improvements of practical importance when it comes to medal eligibility. Therefore, despite the lack of large differences in the different physiological, biomechanical, or perceptive parameters between the BJ and PLA groups, the small effects on some of the monitored parameters could provide key insights into where some marginal gains could be achieved. Some clearer effects of the ergogenic potential of BJ could probably be observed by manipulating the work-to-rest ratios, by comparing swimmers of different expertise levels (Domínguez et al., 2018; Zamani et al., 2020), or even by including biomarkers of inflammation and muscle damage.

## 5 Conclusion

The results of this study indicate that acute ingestion of a dose of BJ 3 h before a 6 × 100-m test at maximum speed did not significantly affect the repeated swimming performance, lactate response, heart rate, or subjective perceived exertion of competitive swimmers. However, some possible positive effects of BJ were observed in the performance of the last repetition, as well as in some indicators of a better recovery, that could affect the marginal gains sought during competitive events. Further research with competitive swimmers in which additional effort conditions could be manipulated should be conducted to further examine these possible beneficial effects of BJ supplementation in swimmers.

## Data Availability

The original contributions presented in the study are included in the article/Supplementary Material, further inquiries can be directed to the corresponding author.
