# Supplementary material for: Effects of beetroot juice intake on repeated performance of competitive swimmers
Source: Front Physiol. 2023 Jan 10;13:1076295. doi: 10.3389/fphys.2022.1076295 (PMC9871287; doi:10.3389/fphys.2022.1076295)
Supplement: Supplementary file 1 [file Table1.DOCX]

| **Table Supplementary 1.** Performance, physiological, kinematical and psychophysiological differences between Beetroot Juice (BJ) and Placebo conditions during a repeated sprint performance by competitive swimmers. | | | | | | | | | | | | | | | | | | | | | | |
| --- | --- | --- | --- | --- | --- | --- | --- | --- | --- | --- | --- | --- | --- | --- | --- | --- | --- | --- | --- | --- | --- | --- |
|  | | | | | | | | | | | | | | | | | | | | | | |
|  |  |  | Beetroot Juice | | |  | Placebo | | |  | Beetroot Juice vs Placebo | | | | | | | | | | | |
|  |  |  | Mean | SD | *p* vs baseline |  | Mean | SD | *p* vs baseline |  | *p* | Change in mean (%) | 90% LL | 90%UL | Cohen´s d | 90% LL | 90%UL | +ive | Trivial | -ive | clinical inference |  |
| **50meters. time (s)** | |  |  |  |  |  |  |  |  |  |  |  |  |  |  |  |  |  |  |  |  |  |
|  | Repetition 1 |  | 30.66 | 2.28 | - |  | 30.60 | 2.40 | - |  | 0.839 | -0.23 | -1.96 | 1.53 | -0.03 | -0.25 | 0.19 | 5 | 85 | 10 | likely trivial |  |
|  | Repetition 2 |  | 30.71 | 2.41 | 0.830 |  | 30.49 | 2.83 | 0.659 |  | 0.456 | -0.83 | -2.60 | 0.98 | -0.10 | -0.32 | 0.12 | 2 | 77 | 22 | likely trivial |  |
|  | Repetition 3 |  | 31.05 | 2.61 | 0.122 |  | 30.95 | 2.23 | 0.078 |  | 0.691 | -0.26 | -1.65 | 1.16 | -0.03 | -0.19 | 0.13 | 1 | 95 | 4 | likely trivial |  |
|  | Repetition 4 |  | 31.23 | 2.36 | 0.024 |  | 30.85 | 1.92 | 0.328 |  | 0.104 | -1.13 | -2.36 | 0.11 | -0.14 | -0.30 | 0.01 | 0 | 74 | 26 | possibly –ive |  |
|  | Repetition 5 |  | 31.51 | 2.50 | 0.009 |  | 31.62 | 2.16 | 0.001 |  | 0.652 | 0.42 | -0.98 | 1.84 | 0.05 | -0.12 | 0.22 | 7 | 92 | 1 | likely trivial |  |
|  | Repetition 6 |  | 31.04 | 2.47 | 0.261 |  | 31.55 | 2.34 | 0.004 |  | 0.112 | 1.67 | -0.12 | 3.50 | 0.20 | -0.01 | 0.41 | 49 | 51 | 0 | possibly +ive |  |
| **100meters. time (s)** | |  |  |  |  |  |  |  |  |  |  |  |  |  |  |  |  |  |  |  |  |  |
|  | Repetition 1 |  | 63.69 | 4.11 | - |  | 64.06 | 5.04 | - |  | 0.600 | 0.49 | -1.41 | 2.41 | 0.07 | -0.21 | 0.35 | 22 | 73 | 6 | Unclear |  |
|  | Repetition 2 |  | 64.13 | 4.85 | 0.287 |  | 64.21 | 4.85 | 0.619 |  | 0.875 | 0.12 | -1.32 | 1.59 | 0.02 | -0.17 | 0.20 | 5 | 92 | 3 | likely trivial |  |
|  | Repetition 3 |  | 64.42 | 5.23 | 0.185 |  | 64.73 | 4.65 | 0.100 |  | 0.608 | 0.55 | -1.02 | 2.15 | 0.07 | -0.12 | 0.25 | 11 | 88 | 1 | likely trivial |  |
|  | Repetition 4 |  | 64.50 | 4.73 | 0.108 |  | 64.72 | 4.28 | 0.147 |  | 0.711 | 0.37 | -1.11 | 1.87 | 0.05 | -0.14 | 0.24 | 9 | 89 | 2 | likely trivial |  |
|  | Repetition 5 |  | 65.20 | 5.06 | 0.014 |  | 65.64 | 3.84 | 0.029 |  | 0.545 | 0.78 | -1.08 | 2.67 | 0.10 | -0.13 | 0.33 | 22 | 76 | 2 | likely trivial |  |
|  | Repetition 6 |  | 64.77 | 4.83 | 0.052 |  | 65.76 | 4.48 | 0.015 |  | 0.104 | 1.57 | -0.04 | 3.20 | 0.20 | -0.01 | 0.40 | 49 | 50 | 0 | possibly +ive |  |
| **Lactate (mmol)** | |  |  |  |  |  |  |  |  |  |  |  |  |  |  |  |  |  |  |  |  |  |
|  | At rest |  | 1.43 | 0.39 | - |  | 1.29 | 0.36 | - |  | 0.343 | -9.68 | -25.13 | 8.95 | -0.34 | -0.97 | 0.29 | 8 | 27 | 65 | Unclear |  |
|  | Repetition 1 |  | 7.66 | 2.85 | <0.001 |  | 8.17 | 1.41 | <0.001 |  | 0.623 | 14.89 | -12.00 | 49.98 | 0.27 | -0.25 | 0.79 | 59 | 34 | 7 | Unclear |  |
|  | Repetition 2 |  | 11.28 | 3.67 | <0.001 |  | 10.71 | 2.27 | <0.001 |  | 0.703 | -3.04 | -23.69 | 23.18 | -0.09 | -0.82 | 0.63 | 24 | 36 | 40 | Unclear |  |
|  | Repetition 3 |  | 11.99 | 4.21 | <0.001 |  | 12.70 | 2.77 | <0.001 |  | 0.680 | 10.80 | -15.48 | 45.25 | 0.24 | -0.39 | 0.88 | 54 | 34 | 12 | Unclear |  |
|  | Repetition 4 |  | 13.00 | 3.94 | <0.001 |  | 13.76 | 3.45 | <0.001 |  | 0.649 | 7.56 | -15.50 | 36.90 | 0.21 | -0.48 | 0.89 | 51 | 34 | 16 | Unclear |  |
|  | Repetition 5 |  | 13.26 | 4.25 | <0.001 |  | 13.55 | 3.41 | <0.001 |  | 0.875 | 5.12 | -18.88 | 36.21 | 0.12 | -0.52 | 0.77 | 42 | 39 | 19 | Unclear |  |
|  | Repetition 6 |  | 14.76 | 4.14 | <0.001 |  | 13.66 | 2.31 | <0.001 |  | 0.385 | -4.82 | -19.50 | 12.54 | -0.15 | -0.65 | 0.35 | 12 | 45 | 43 | Unclear |  |
| **Stroke rate (rpm)** | |  |  |  |  |  |  |  |  |  |  |  |  |  |  |  |  |  |  |  |  |  |
|  | Repetition 1 |  | 45.16 | 3.89 | - |  | 44.22 | 3.91 | - |  | 0.500 | -2.10 | -7.14 | 3.22 | -0.23 | -0.80 | 0.34 | 10 | 36 | 54 | Unclear |  |
|  | Repetition 2 |  | 44.40 | 4.22 | 0.443 |  | 44.37 | 4.08 | 0.879 |  | 0.977 | -0.04 | -4.52 | 4.65 | 0.00 | -0.45 | 0.44 | 21 | 56 | 22 | Unclear |  |
|  | Repetition 3 |  | 44.23 | 3.70 | 0.377 |  | 43.54 | 3.18 | 0.427 |  | 0.454 | -1.49 | -5.09 | 2.25 | -0.17 | -0.58 | 0.25 | 7 | 49 | 44 | Unclear |  |
|  | Repetition 4 |  | 44.47 | 3.93 | 0.515 |  | 43.77 | 3.77 | 0.461 |  | 0.509 | -1.55 | -5.67 | 2.75 | -0.16 | -0.61 | 0.28 | 9 | 47 | 44 | Unclear |  |
|  | Repetition 5 |  | 44.25 | 3.45 | 0.371 |  | 44.20 | 4.30 | 0.986 |  | 0.954 | -0.29 | -3.91 | 3.46 | -0.04 | -0.48 | 0.41 | 18 | 55 | 26 | Unclear |  |
|  | Repetition 6 |  | 44.59 | 3.84 | 0.628 |  | 43.58 | 4.41 | 0.502 |  | 0.205 | -2.40 | -5.45 | 0.74 | -0.27 | -0.62 | 0.08 | 2 | 35 | 64 | possibly –ive |  |
| **Rate of Perceived Exertion (AU)** | |  |  |  |  |  |  |  |  |  |  |  |  |  |  |  |  |  |  |  |  |  |
|  | Repetition 1 |  | 13.85 | 2.67 | - |  | 14.69 | 1.75 | - |  | 0.242 | 7.22 | -2.10 | 17.44 | 0.33 | -0.10 | 0.77 | 70 | 27 | 2 | possibly +ive |  |
|  | Repetition 2 |  | 15.23 | 2.56 | 0.002 |  | 16.00 | 1.68 | 0.007 |  | 0.165 | 5.96 | -0.80 | 13.17 | 0.31 | -0.04 | 0.65 | 70 | 29 | 1 | possibly +ive |  |
|  | Repetition 3 |  | 16.31 | 1.89 | <0.001 |  | 16.77 | 1.79 | 0.001 |  | 0.363 | 2.89 | -2.65 | 8.75 | 0.23 | -0.22 | 0.68 | 55 | 40 | 6 | Unclear |  |
|  | Repetition 4 |  | 17.15 | 1.35 | <0.001 |  | 17.62 | 1.61 | <0.001 |  | 0.291 | 2.56 | -1.92 | 7.24 | 0.30 | -0.23 | 0.83 | 63 | 31 | 6 | Unclear |  |
|  | Repetition 5 |  | 17.31 | 2.53 | 0.007 |  | 17.77 | 1.83 | <0.001 |  | 0.624 | 3.39 | -7.30 | 15.32 | 0.18 | -0.40 | 0.76 | 47 | 39 | 13 | Unclear |  |
|  | Repetition 6 |  | 18.69 | 1.03 | <0.001 |  | 18.77 | 1.48 | <0.001 |  | 0.856 | 0.25 | -3.73 | 4.41 | 0.04 | -0.64 | 0.72 | 34 | 39 | 27 | Unclear |  |
| **Total Quality Recovery Scale (AU)** | |  |  |  |  |  |  |  |  |  |  |  |  |  |  |  |  |  |  |  |  |  |
|  | Prior to repetition 2 |  | 13.08 | 1.94 | - |  | 11.92 | 1.55 | - |  | 0.110 | -8.54 | -17.08 | 0.88 | -0.53 | -1.12 | 0.05 | 2 | 14 | 83 | likely –ive |  |
|  | Prior to repetition 3 |  | 11.85 | 2.30 | 0.032 |  | 11.08 | 1.44 | 0.027 |  | 0.268 | -5.44 | -14.53 | 4.62 | -0.25 | -0.71 | 0.20 | 5 | 37 | 58 | Unclear |  |
|  | Prior to repetition 4 |  | 11.00 | 2.35 | 0.006 |  | 9.85 | 1.68 | 0.004 |  | 0.082 | -9.74 | -18.36 | -0.22 | -0.44 | -0.87 | -0.01 | 1 | 16 | 83 | likely –ive |  |
|  | Prior to repetition 5 |  | 10.85 | 3.29 | 0.051 |  | 10.08 | 2.50 | 0.109 |  | 0.516 | -5.48 | -21.48 | 13.78 | -0.17 | -0.73 | 0.39 | 13 | 41 | 46 | Unclear |  |
|  | Prior to repetition 6 |  | 9.15 | 2.44 | <0.001 |  | 9.00 | 1.68 | <0.001 |  | 0.846 | -0.07 | -14.36 | 16.61 | 0.00 | -0.54 | 0.53 | 26 | 48 | 26 | Unclear |  |
| **Heart rate max (bpm)** | |  |  |  |  |  |  |  |  |  |  |  |  |  |  |  |  |  |  |  |  |  |
|  | 1 minute |  | 141.77 | 23.21 | - |  | 151.38 | 24.24 | - |  | 0.302 | 7.05 | -3.88 | 19.22 | 0.36 | -0.21 | 0.93 | 69 | 26 | 5 | Unclear |  |
|  | 3 minutes |  | 109.08 | 13.80 | <0.001 |  | 101.31 | 40.02 | 0.001 |  | 0.494 | -19.87 | -43.84 | 14.35 | -1.66 | -4.32 | 1.00 | 12 | 6 | 83 | Unclear |  |
| **Caunter Movement Jump (cm)** | |  |  |  |  |  |  |  |  |  |  |  |  |  |  |  |  |  |  |  |  |  |
|  | Pre |  | 25.57 | 3.54 | - |  | 26.88 | 6.16 | - |  | 0.326 | 3.69 | -4.76 | 12.90 | 0.25 | -0.34 | 0.84 | 56 | 34 | 10 | Unclear |  |
|  | Post |  | 25.48 | 5.11 | 0.909 |  | 25.72 | 6.00 | 0.013 |  | 0.767 | 0.24 | -5.57 | 6.41 | 0.01 | -0.30 | 0.33 | 15 | 72 | 12 | Unclear |  |
| **Underwater distance start (m)** | |  |  |  |  |  |  |  |  |  |  |  |  |  |  |  |  |  |  |  |  |  |
|  | Repetition 1 |  | 8.10 | 0.74 | - |  | 7.76 | 0.88 | - |  | 0.034 | -4.33 | -7.27 | -1.29 | -0.44 | -0.75 | -0.13 | 0 | 10 | 90 | likely –ive |  |
|  | Repetition 2 |  | 8.18 | 0.82 | 0.530 |  | 8.10 | 1.09 | 0.072 |  | 0.790 | -1.29 | -7.15 | 4.94 | -0.12 | -0.67 | 0.44 | 16 | 44 | 40 | Unclear |  |
|  | Repetition 3 |  | 8.35 | 0.95 | 0.189 |  | 8.27 | 0.97 | <0.001 |  | 0.667 | -1.01 | -5.00 | 3.14 | -0.08 | -0.42 | 0.25 | 8 | 65 | 27 | Unclear |  |
|  | Repetition 4 |  | 8.58 | 0.99 | 0.009 |  | 8.21 | 0.80 | <0.001 |  | 0.014 | -4.22 | -6.72 | -1.64 | -0.34 | -0.55 | -0.13 | 0 | 13 | 87 | likely –ive |  |
|  | Repetition 5 |  | 8.23 | 1.06 | 0.619 |  | 8.19 | 0.92 | 0.002 |  | 0.888 | -0.30 | -5.84 | 5.58 | -0.02 | -0.44 | 0.40 | 18 | 59 | 23 | Unclear |  |
|  | Repetition 6 |  | 8.35 | 1.20 | 0.358 |  | 8.19 | 0.93 | 0.028 |  | 0.449 | -1.53 | -5.67 | 2.79 | -0.10 | -0.39 | 0.18 | 4 | 68 | 28 | possibly –ive |  |
| **Underwater distance turn (m)** | |  |  |  |  |  |  |  |  |  |  |  |  |  |  |  |  |  |  |  |  |  |
|  | Repetition 1 |  | 4.22 | 0.38 | - |  | 4.16 | 0.42 | - |  | 0.470 | -1.61 | -5.11 | 2.02 | -0.17 | -0.55 | 0.21 | 5 | 50 | 45 | Unclear |  |
|  | Repetition 2 |  | 4.09 | 0.42 | 0.049 |  | 4.05 | 0.47 | 0.138 |  | 0.760 | -1.00 | -5.62 | 3.85 | -0.09 | -0.54 | 0.35 | 13 | 53 | 34 | Unclear |  |
|  | Repetition 3 |  | 4.19 | 0.56 | 0.750 |  | 4.13 | 0.37 | 0.775 |  | 0.659 | -1.11 | -6.71 | 4.81 | -0.08 | -0.51 | 0.35 | 13 | 55 | 32 | Unclear |  |
|  | Repetition 4 |  | 4.15 | 0.38 | 0.277 |  | 4.05 | 0.44 | 0.281 |  | 0.179 | -2.55 | -5.57 | 0.55 | -0.27 | -0.59 | 0.06 | 1 | 35 | 64 | possibly –ive |  |
|  | Repetition 5 |  | 4.17 | 0.40 | 0.550 |  | 4.03 | 0.33 | 0.336 |  | 0.288 | -3.13 | -8.38 | 2.42 | -0.30 | -0.82 | 0.22 | 6 | 32 | 63 | Unclear |  |
|  | Repetition 6 |  | 3.98 | 0.41 | 0.002 |  | 3.98 | 0.43 | 0.134 |  | 0.948 | -0.16 | -4.07 | 3.91 | -0.01 | -0.37 | 0.34 | 15 | 66 | 19 | Unclear |  |
| **Underwater velocity start (m/s)** | |  |  |  |  |  |  |  |  |  |  |  |  |  |  |  |  |  |  |  |  |  |
|  | Repetition 1 |  | 2.71 | 0.20 | - |  | 2.82 | 0.32 | - |  | 0.149 | 3.68 | -0.91 | 8.49 | 0.47 | -0.12 | 1.06 | 79 | 18 | 3 | likely +ive |  |
|  | Repetition 2 |  | 2.75 | 0.23 | 0.39 |  | 2.77 | 0.30 | 0.281 |  | 0.686 | 0.70 | -3.20 | 4.75 | 0.08 | -0.36 | 0.51 | 31 | 55 | 14 | Unclear |  |
|  | Repetition 3 |  | 2.72 | 0.23 | 0.75 |  | 2.76 | 0.25 | 0.154 |  | 0.337 | 1.17 | -0.98 | 3.36 | 0.13 | -0.11 | 0.37 | 30 | 68 | 1 | possibly +ive |  |
|  | Repetition 4 |  | 2.67 | 0.19 | 0.52 |  | 2.72 | 0.23 | 0.062 |  | 0.248 | 1.66 | -0.86 | 4.24 | 0.21 | -0.11 | 0.54 | 53 | 45 | 2 | possibly +ive |  |
|  | Repetition 5 |  | 2.73 | 0.32 | 0.74 |  | 2.70 | 0.31 | 0.038 |  | 0.664 | -1.20 | -6.54 | 4.46 | -0.09 | -0.51 | 0.33 | 12 | 55 | 33 | Unclear |  |
|  | Repetition 6 |  | 2.72 | 0.31 | 0.88 |  | 2.70 | 0.24 | 0.098 |  | 0.772 | -0.34 | -3.94 | 3.41 | -0.03 | -0.33 | 0.28 | 10 | 73 | 17 | Unclear |  |
| **Underwater velocity turn (m/s)** | |  |  |  |  |  |  |  |  |  |  |  |  |  |  |  |  |  |  |  |  |  |
|  | Repetition 1 |  | 2.20 | 0.18 | - |  | 2.23 | 0.21 | - |  | 0.601 | 1.21 | -2.74 | 5.33 | 0.14 | -0.32 | 0.60 | 41 | 49 | 11 | Unclear |  |
|  | Repetition 2 |  | 2.21 | 0.17 | 0.776 |  | 2.26 | 0.18 | 0.216 |  | 0.264 | 2.61 | -1.21 | 6.58 | 0.32 | -0.15 | 0.79 | 67 | 29 | 4 | possibly +ive |  |
|  | Repetition 3 |  | 2.28 | 0.20 | 0.051 |  | 2.26 | 0.17 | 0.413 |  | 0.726 | -0.74 | -4.65 | 3.33 | -0.08 | -0.51 | 0.35 | 13 | 55 | 31 | Unclear |  |
|  | Repetition 4 |  | 2.25 | 0.20 | 0.065 |  | 2.22 | 0.20 | 0.934 |  | 0.370 | -1.44 | -4.06 | 1.25 | -0.16 | -0.45 | 0.14 | 3 | 57 | 40 | possibly –ive |  |
|  | Repetition 5 |  | 2.25 | 0.19 | 0.174 |  | 2.24 | 0.20 | 0.660 |  | 0.882 | -0.25 | -2.79 | 2.36 | -0.03 | -0.31 | 0.26 | 9 | 76 | 15 | Unclear |  |
|  | Repetition 6 |  | 2.27 | 0.18 | 0.135 |  | 2.29 | 0.19 | 0.256 |  | 0.685 | 0.68 | -2.16 | 3.59 | 0.08 | -0.26 | 0.42 | 27 | 65 | 8 | Unclear |  |
